# THE ROLE OF COMMUNITY COLLEGES IN TRAINING HEALTH CARE WORKERS FOR AN AGING POPULATION

**DOI:** 10.1093/geroni/igad104.1688

**Published:** 2023-12-21

**Authors:** Phyllis Cummins, Philip Taylor

## Abstract

The aging population in the U.S. has resulted in an increased need for healthcare workers in multiple health settings (e.g., home healthcare, nursing homes, hospitals, medical laboratories). Community colleges play an important role in educating healthcare workers that are critical to an aging population. Many of these occupations focus on home healthcare, empowering adults to remain at home rather than in a long-term care setting. This session will focus on novel ways that community colleges are effectively addressing workforce needs in multiple settings, including hospitals, home healthcare, long-term care, and medical laboratories. A panel of community college and university gerontology and education professionals representing GSA’s Community College and the Aging Workforce Interest Groups will share the innovative ways in which they are addressing the needs of an aging population. Yamashita and Narine will discuss the current and future demand for employees in healthcare occupations, especially those in long-term and home healthcare settings, and will focus on careers that do not require a bachelor’s degree. Velazquez’s presentation will focus on the impact COVID had on training healthcare workers in healthcare related occupations and how one community colleges adapted their curriculum. Cummins et al. will discuss a study that focused on the role literacy, numeracy, and problem-solving skills play in community college students’ success in the classroom and the healthcare workplace, focusing on employer interviews. Finally, Kaur will discuss the role Hillsborough Community College plays in training medical laboratory scientists and in providing employees for critical medical services to an aging society This is a collaborative symposium between the Aging Workforce and Community College Interest Groups.

